# Taking Constructivism One Step Further: Post Hoc Analysis of a Student-Created Wiki

**DOI:** 10.2196/mededu.9197

**Published:** 2018-06-14

**Authors:** Michael Pascoe, Forrest Monroe, Helen Macfarlane

**Affiliations:** ^1^ Physical Therapy Program School of Medicine University of Colorado Anschutz Medical Campus Aurora, CO United States; ^2^ Modern Human Anatomy Program Department of Cell and Development Biology University of Colorado Anschutz Medical Campus Aurora, CO United States; ^3^ Department of Anesthesiology School of Medicine University of Kansas Medical Center Kansas City, CO United States; ^4^ School of Medicine University of Colorado Anschutz Medical Campus Aurora, CO United States

**Keywords:** wiki, constructivist learning, medical education, analytics

## Abstract

**Background:**

Wiki platform use has potential to improve student learning by improving engagement with course material. A student-created wiki was established to serve as a repository of study tools for students in a medical school curriculum. There is a scarcity of information describing student-led creation of wikis in medical education.

**Objective:**

The aim is to characterize website traffic of a student-created wiki and evaluate student perceptions of usage via a short anonymous online survey.

**Methods:**

Website analytics were used to track visitation statistics to the wiki and a survey was distributed to assess ease of use, interest in contributing to the wiki, and suggestions for improvement.

**Results:**

Site traffic data indicated high usage, with a mean of 315 (SD 241) pageviews per day from July 2011 to March 2013 and 74,317 total user sessions. The mean session duration was 1.94 (SD 1.39) minutes. Comparing Fall 2011 to Fall 2012 sessions revealed a large increase in returning visitors (from 12,397 to 20,544, 65.7%) and sessions via mobile devices (831 to 1560, 87.7%). The survey received 164 responses; 88.0% (162/184) were aware of the wiki at the time of the survey. On average, respondents felt that the wiki was more useful in the preclinical years (mean 2.73, SD 1.25) than in the clinical years (mean 1.88, SD 1.12; *P*<.001). Perceived usefulness correlated with the percent of studying for which the respondent used electronic resources (Spearman ρ=.414, *P*<.001).

**Conclusions:**

Overall, the wiki was a highly utilized, although informal, part of the curriculum with much room for improvement and future exploration.

## Introduction

### Wiki Use and Underlying Educational Theory

Wikis belong to a broader class of “Web 2.0” online tools, which draw from social engagement of users to directly create and modify content, rather than the traditional model of publisher to consumer. Wikipedia (Wikimedia Foundation Inc, San Francisco, CA, USA) is the best-known wiki website. Web 2.0 tools and wikis have become very common not only among medical students, but also among physicians. One UK survey found that more than 80% of junior physicians used Web 2.0 tools for professional purposes, with wikis being the most common [1].

**Figure 1:**
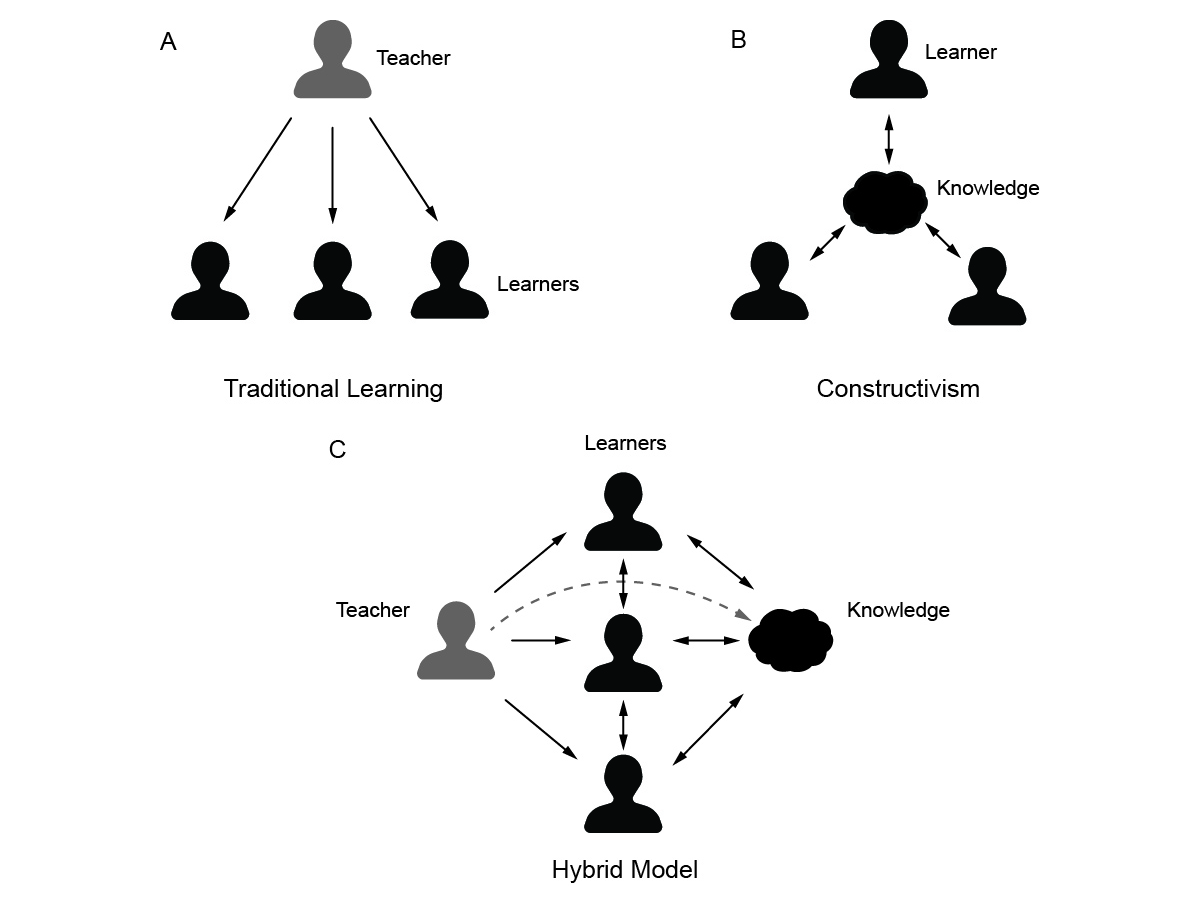
Models of learning in medical education. A wiki can serve as a hybrid model (C) between the traditional (A) and constructivist (B) learning environments by enabling learners to self-organize knowledge they receive from the teacher. The broken line in the hybrid model (C) represents the ability of the teacher to contribute and review information in the knowledge base.

Wikis are supported by the constructivist model of learning, which states that students learn best by actively creating their own knowledge structures, in contrast to traditional behaviorism models in which education is a unidirectional flow of information from teacher to learner [2,3]. Wikis can take constructivism one step further by allowing students to collaboratively create and organize structures of knowledge. In constructivism, the entire learning process is self-directed by the learner. Wikis are a valuable supplement to classroom learning because they allow students to reformulate knowledge, integrate concepts across multiple lectures, and assure their understanding [4]. The potential for students to interact with one another through the wiki serves both a social and educational role. Students can create a “Folksonomy” (folk taxonomy) of information by tagging useful websites and resources for their peers, as opposed to a typical taxonomy of information bestowed in a top-down fashion from a lecturer or textbook [5]. Wikis can serve as part of a hybrid model between the traditional and constructivist learning environments by enabling students to self-organize and augment knowledge they receive from top-down teaching methods (Figure 1). Because teachers are also free to interact with a wiki, they too can participate by guiding the structure and content. Wikis can be powerful in the context of a course because they allow students to build on work by previous learners, creating a robust and refined document. In addition, wikis can provide task-specific benefits: when wikis were utilized for group work in a nursing program, students felt that it helped build knowledge, monitor progress, and avoid redundant work [6]. Because any user can edit any content, the wiki model allows those with the passion and knowledge to contribute to their colleagues’ education. In addition, work is instantly subjected to an informal and perpetual peer-review process.

**Figure 2:**
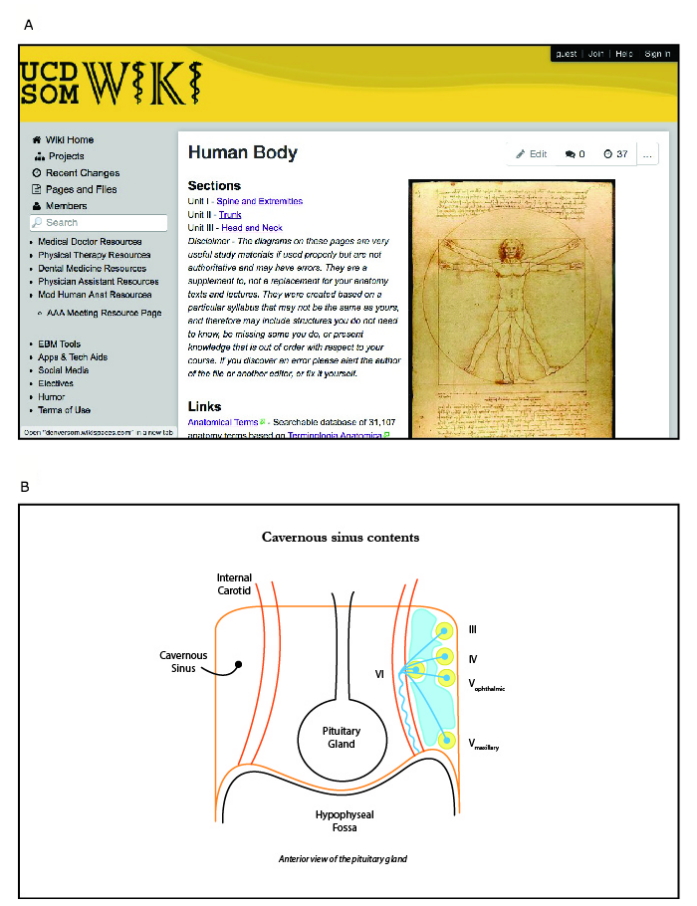
Screenshots showing an example page of a curricular block (A) and content (B).

### Wikis in Medical Education

Smaller and more focused wikis are becoming popular in many educational settings due to the collaboration and knowledge sharing they support. A wide variety of uses for wikis in education have been documented in the literature, including collectively annotating class reading, publishing syllabi and other class documents, concept mapping, resource sharing, and group authoring of documents [7]. Many researchers have published specifically within medical education and concluded they have significant potential and require further study [8,9]. In addition, several medical school and graduate medical education residency program wikis are documented to contain large quantities of content with a large volume of Web traffic. For example, a medical student-initiated wiki had nearly 1600 pages and 1.2 million page views covering most aspects of their curriculum [10]. Other medical schools have utilized wikis in a more focused form. An elective at one medical school had students write brief appraisals of evidence that they then placed on Wikipedia; course reviews were favorable [11]. A pathology residency program incorporated a wiki into a course for second- and third-year residents by asking participants to write online review articles based on assigned lectures. Pretest and posttest data indicated a greater increase in test scores compared to previous years (25% versus 16%) [12]. A large internal medicine residency program incorporated a wiki to share frequently accessed links such as forms and contact information as well as brief summaries and links to curated websites with more authoritative information [13]. Their survey showed that 100% of house staff felt the wiki improved their ability to complete tasks, 89% reported it improved their efficiency, and 57% reported it improved their education. In another example, an anesthesiology department utilized a wiki for guiding residents to educational materials such as podcasts and lectures [14]. Wikis also have the potential to promote collaboration more broadly within a specialty. For example, OpenAnesthesia.org is a large and growing wiki that covers many anesthesia topics, and received more than 10,000 visitors in its first month of operation [15]. The results of these studies demonstrate the primary strengths of wikis: to hold useful, well-organized information in an easily updated format.

### Wiki Establishment and Growth

The preclinical curriculum of the University of Colorado (first two years; MS1 and MS2) is composed of organ system-based blocks, except for the first block, Human Body block (gross anatomy). Early in the 2009-2010 academic year, students of the Class of 2013 began using Google Groups (Google Inc, Mountain View, CA, USA) to share files and Web links such as electronic flashcard sets and useful websites. However, the files were stored in a single list that quickly grew to hundreds of entries and included irrelevant or outdated items. In addition, the files were part of the Class of 2013 Google Group, which were not accessible to other classes. A wiki website was chosen because it offered an easy way for all classes to contribute in a format that most students would be familiar with due to the popularity of Wikipedia.

The block structure of the medical school curriculum served as the template for organizing wiki content, with pages for each block and subpages within each block section (eg, Human Body, Figure 2A). The wiki platform Wikispaces (Tangient LLC, San Francisco, CA, USA) was selected as the host of the wiki and a URL was established [16]. A handful of students from each successive class began posting additional material. This level of engagement is typical of wikis in which participation is not required, as suggested by a study of Wikipedia that found 1% of users make half of the page edits [17]. The wiki received an additional boost in content from an anatomy instructor (MAP), who contributed many diagrams (eg, the cavernous sinus, Figure 2B).

Although the benefits of wiki use in medical education have been documented, there is a paucity of information on the utilization and perceived value of a student-led wiki. Therefore, the aims of this study were to (1) quantify the utilization of a student-led wiki through website traffic analysis and (2) evaluate the perceptions of usage through individual survey of medical students at our institution. We hypothesized that the wiki would be highly utilized by medical students and perceived as useful in their studies.

## Methods

### Study Participants

The University of Colorado Anschutz Medical Campus (Aurora, CO, USA) is a large academic medical center that includes programs in medicine, dentistry, pharmacy, nursing, physical therapy, and physician assistant studies. At the time of the study, the medical school enrolled approximately 160 students per class year.

### Wiki Traffic Analysis

The utilization of the University of Colorado School of Medicine (CUSOM) wiki was quantified using Google Analytics (Google Inc, Mountain View, CA, USA), an open-source tool for collecting detailed website traffic information, which was added to the wiki in July 2011. Google Analytics defines a session as a group of interactions within a given time frame. A session was considered terminated once there was 30 minutes of inactivity. New visitors were logged specific to the browser and device that was used. A pageview was an instance of a page being loaded (or reloaded) in a browser. To gain a sense of how long users were engaging with a page of the wiki, average session duration was calculated by dividing the total duration of all sessions (in seconds) by the number of sessions. To determine degree of engagement with content, bounce rate was calculated as the percentage of single-page sessions (ie, sessions a user left the wiki from the entrance page without interacting with the page). To assess changes over time, a comparison of website traffic was performed between the Fall 2011 and Fall 2012 semesters. It is important to acknowledge the potential influence of fake visits on Web traffic for any website employing Google Analytics (ie, ghost spam). To quantify the size of this effect, we counted and subsequently filtered out the sessions originating from hostnames outside of the wikispaces subdomain [16].

To gain insight into device usage patterns, sessions were categorized by type (desktop/laptop, tablet, or mobile). The pages with the most pageviews were also determined in order to understand what resources were most utilized.

### Usage Survey

To obtain individual-level data about medical student use of the wiki, a survey was developed online using Google Forms (Google, Inc). After technical functionality of the survey was tested, the survey URL was distributed by email to a convenience sample of all four current classes in the School of Medicine (N=640; classes of 2013-2016), with one follow-up email sent as a reminder. The survey was open for 2 weeks (January 2-16, 2013). The survey (Multimedia Appendix 1) consisted of 20 questions, nine of which all respondents were presented with and an additional 11 that were presented only if the student indicated past wiki use. The survey assessed students’ user experience with the wiki, including ease of navigation, satisfaction with content, and importance as part of their education. Students were also asked about self-perceived willingness and ability to edit or make contributions to the wiki. Items on the survey were formatted as choose from a list, 5-point Likert evaluation scale, or open field response. Individual student submissions were collated in a Google Spreadsheet and later downloaded to a Microsoft Excel spreadsheet (Microsoft Corp, Redmond, WA) for in-depth analysis and data cleaning. Only completed questionnaires were analyzed.

Descriptive summary statistics were calculated for close-ended usage survey questions. The Kruskal-Wallis test (nonparametric) was used to analyze differences in the six Likert scale questions of the survey (see Multimedia Appendix 1) across the groups (ie, student class year). The degree of linear association between two survey items was examined using Spearman rho rank correlation coefficient. Positive rho values between zero and .2 indicated no correlation. Values between .2 and .5 represented a weak correlation; between .5 and .8, a moderate correlation; and greater than .8, a strong to perfect correlation [18].

An alpha level of *P*<.05 was used to identify significant differences and statistical analyses were performed using SPSS version 25 (IBM, Armonk, NY, USA). Data are presented in the text and tables as mean and standard deviation and in figures as mean and standard error. These experimental procedures were reviewed and approved by the Colorado Multiple Institutional Review Board.

## Results

### Wiki Traffic

**Figure 3:**
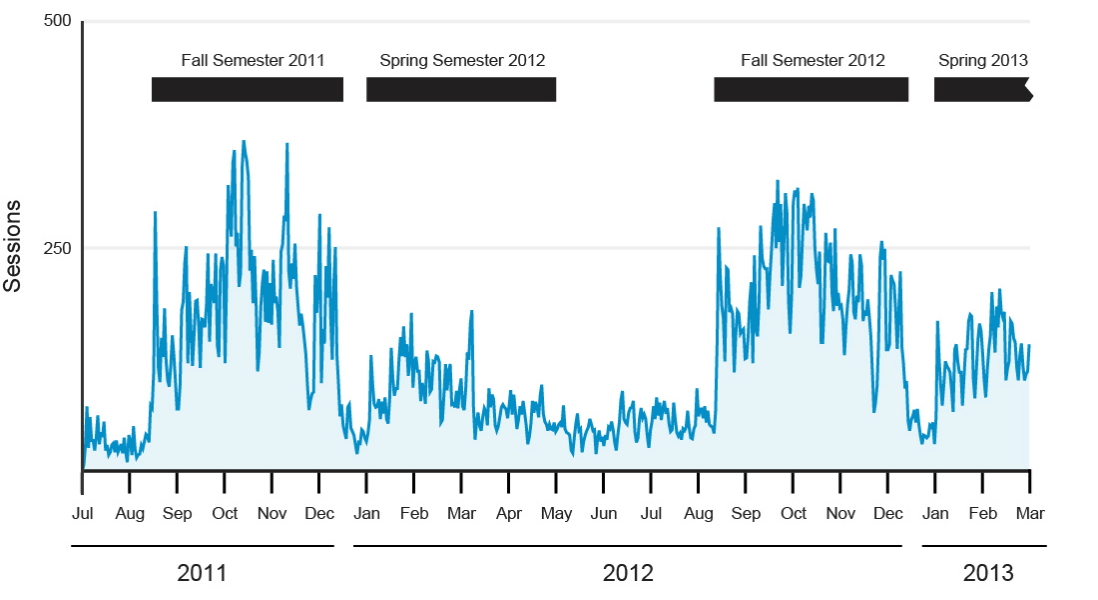
Trend in user sessions compared with timing of academic semesters.

Quantitative data from Google Analytics were collected from July 1, 2011 to March 1, 2013 (20 months; 610 days). During this time, there were a total of 74,317 sessions (daily range: 7-368; mean 122, SD 80 sessions per day), of which 66.93% (49,741) were logged by returning visitors. During these sessions, a total of 192,545 pageviews were generated (mean 315, SD 241 pageviews per day), yielding mean 2.59 pages per session. Most sessions consisted of visiting one page of the wiki (ie, bounce rate; 46,125/74,317 sessions, 62.07%) with 6902 (9.29%) visiting two and 6582 (8.86%) three pages, respectively. The mean session duration was 1.94 (SD 1.39) minutes. There was a noticeable elevation in sessions corresponding to the occurrence of semesters (Figure 3). The number of sessions identified as ghost spam and removed from analysis was very small (8/74,317 sessions, 0.01%).

The type of device used to access the wiki most frequently was a desktop/laptop computer (69,968/74,317 sessions, 94.15%). Tablet and mobile devices accounted for 2355 (3.17%) and 1994 (2.68%) sessions, respectively. The page of the wiki site with the most pageviews was the home page (32,532/192,545, 16.90%), followed by the Head & Neck section of Human Body block (21,106/192,545, 10.96%) and the main page of the Human Body block content (10,076/192,545, 5.23%).

Key website traffic measures were compared between Fall 2011 (August 15 to December 14; 122 days) and Fall 2012 (August 14 to December 15; 124 days) semesters. These data are summarized in Table 1.

**Table 1:** Comparison of website traffic measures between Fall 2011 and Fall 2012 semesters.

Variable	Fall semester 2011	Fall semester 2012	Percent change^a^, %
Sessions, n	22,992	24,861	8.13
Sessions per day, mean (SD)	188.46 (71.24)	200.49 (60.85)	6.38
Returning visitors, n (%)	12,397 (53.92)	20,544 (82.64)	65.72
New visitors, n (%)	10,595 (46.08)	4317 (17.36)	–59.25
Pageviews, n	57,926	70,009	20.86
Pageviews per day, mean (SD)	474.80 (240.54)	564.59 (213.92)	18.91
Pages per session, mean (SD)	2.43 (0.53)	2.79 (0.51)	14.81
Session duration (minutes), mean (SD)	2.20 (1.58)	2.60 (1.21)	18.18
Bounce rate, %	65.04	56.22	–13.6
Sessions via desktop/laptop, n	22,161	23,301	5.1
Sessions via smartphone/tablet, n	831	1560	87.7

^a^Percentage change calculated using (Fall 2012–Fall 2011)/Fall 2011 * 100.

**Table 2:** Summary of medical student responses to the survey.

Variable	Class of 2013	Class of 2014	Class of 2015	Class of 2016	Total	*P* value
Number of responses, n	73	57	19	15	164	—
Response rate, %	46	36	12	9	26	—
Aware of wiki before survey, n (%)	62 (85)	49 (86)	19 (100)	15 (100)	145 (88)	—
Have edited wiki in the past, n (%)	16 (22)	14 (25)	6 (32)	0 (0)	36 (22)	—
Importance in preclinical years^a^, mean (SD)	2.11 (1.08)	2.77 (1.13)	3.74 (0.99)	3.80 (0.94)	2.73 (1.25)	<.001
Importance in clinical years^a^, mean (SD)	1.63 (0.90)	1.75 (1.06)	3.71 (0.76)	3.40 (1.14)	1.88 (1.12)	<.001
Ease of finding content^a^, mean (SD)	3.55 (1.03)	3.51 (0.94)	4.16 (0.77)	4.07 (0.88)	3.68 (0.98)	.0173
Willingness to contribute content^a^, mean (SD)	2.82 (1.22)	2.84 (1.18)	3.00 (1.11)	3.80 (0.94)	2.94 (1.19)	.0292
Ease of making changes^a^, mean (SD)	3.60 (1.27)	3.50 (1.38)	2.67 (0.58)	2.00 (1.41)	3.29 (1.27)	.322
Confidence of adding content^a^, mean (SD)	2.86 (1.41)	2.88 (1.21)	2.89 (1.29)	3.33 (1.45)	2.91 (1.33)	.705

^a^For this survey item, a five-point Likert scale was used with 1=not important at all and 5=essential.

### Usage Survey

Surveys were completed by 164 of 640 students in the four medical school classes (25.6% response rate; Table 2). Overall, 145 of 164 respondents (88.4%) were aware of the CUSOM wiki prior to taking the survey. Among these students, 108 of 145 (74.5%) had heard about it from a classmate, 23 (15.9%) from someone in another class year, and 3 (2.1%) from a professor or administrator. Eleven (7.6%) did not remember how they learned about the wiki. A relatively small number of students had edited the wiki in the past (36/164, 22.0%). The Likert ratings significantly differed between groups for the importance of the wiki in preclinical and clinical years, ease of finding content, and for willingness to contribute. No significant differences were observed for ease of making changes and confidence of adding content.

**Figure 4:**
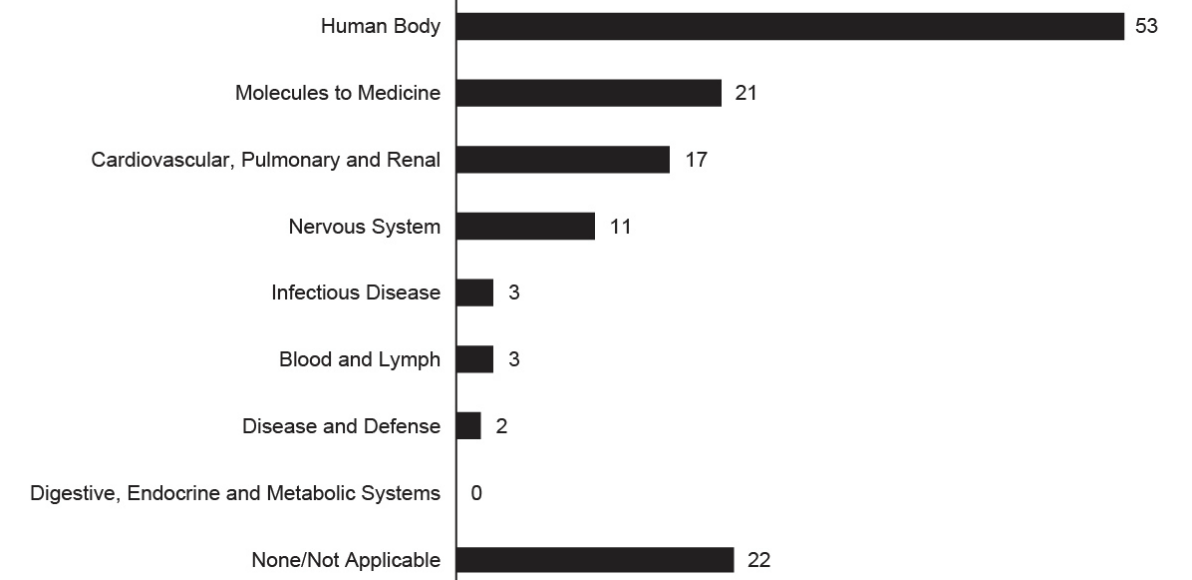
Frequency of reported use of the wiki per block of the medical school curriculum.

Students reported using the wiki the most during the Human Body block of the preclinical years of the curriculum (58/164, 35.4%), whereas 21 and 17 respondents, respectively, used it most for the Molecules to Medicine and the Cardiovascular, Pulmonary, and Renal blocks (Figure 4). Twenty-two students selected none/not applicable for this item.

**Figure 5:**
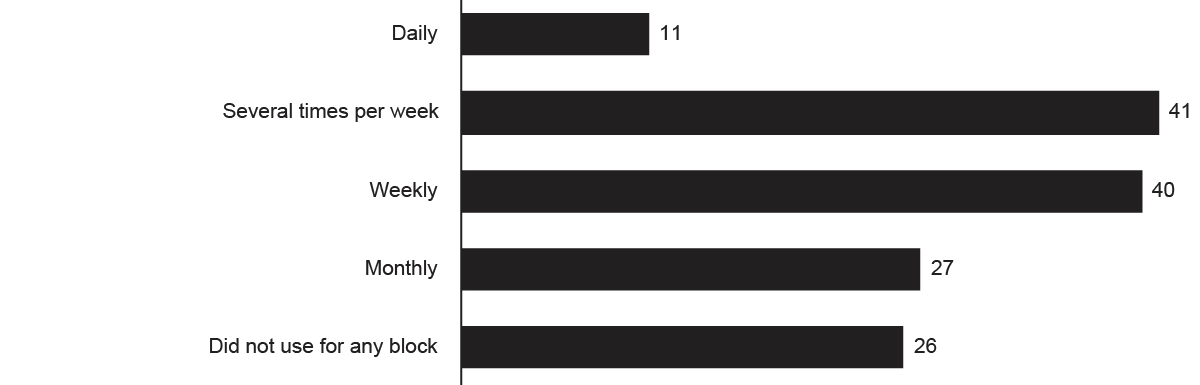
Reported frequency of usage of the wiki.

Students reported content was easy to find on the wiki (mean 3.68, SD 0.98 out of 5.00; N=132). Relatively few students accessed the wiki daily in the block in which they used it most (11/145, 7.6%; Figure 5). A much greater number of students indicated that they accessed the wiki at least once a week (81/145, 55.8%).

**Figure 6:**
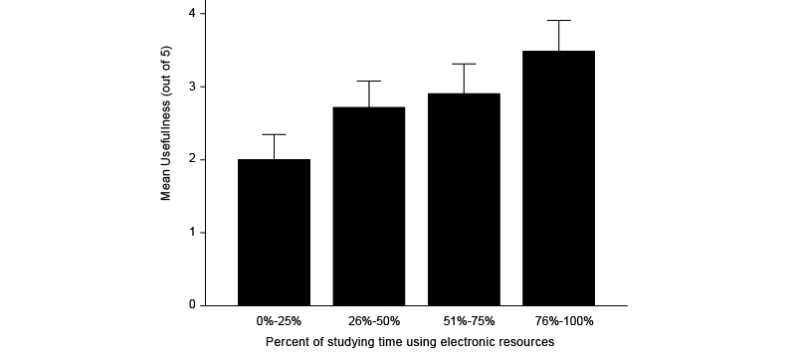
Student-perceived usefulness as a function of percent of studying time using electronic resources. The respondents that used the wiki for a high percentage of time found it more useful (*P*<.001). Error bars represent standard error.

On average, respondents felt the wiki was more useful in the preclinical years of MS1 and MS2 (mean 2.73, SD 1.25, N=143) than in the clinical years of MS3 and MS4 (mean 1.88, SD 1.12, N=120; *P*<.001) on a 5-point scale in which 1 indicated not important at all and 5 indicated essential. Usefulness of the wiki in the preclinical years demonstrated a positive, but weak, correlation with the percent of studying time in the preclinical years using electronic study resources (Spearman ρ=.414, *P*<.001; Figure 6).

**Figure 7:**
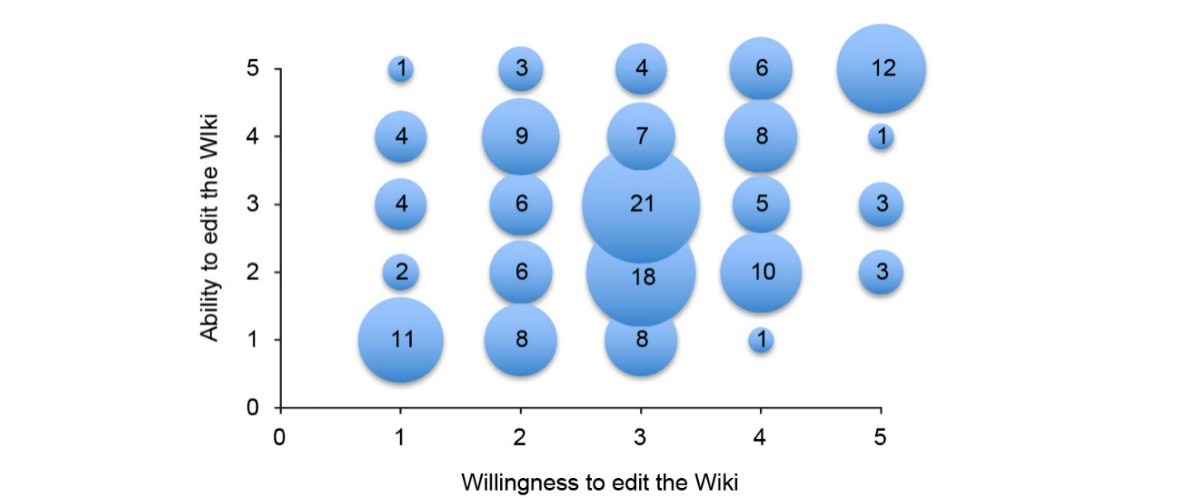
Ability to edit the wiki as a function of willingness to edit the wiki. The numbers in each circle represent the number of respondents.

Level of engagement with editing the wiki was assessed numerically along two dimensions with two Likert scale questions: “How willing will you be to contribute content to the wiki in the future?” and “How confident are you that you could add content if you wanted to?” Average willingness and average confidence were mean 2.94 (SD 1.19) and mean 2.91 (SD 1.33) out of 5, respectively. The most common answer was 3 to both questions (N=162). The answers were weakly correlated (Spearman ρ=.346, *P*<.001). All possible answer pairs were observed except a willingness of 5 and ability of 1 (Figure 7).

## Discussion

### Principal Findings

A collaborative wiki containing valuable study resources for a medical school curriculum was established by students in a relatively short time frame. These results address a gap in the literature on a student-created wiki representing a hybrid model between traditional and constructivist learning theories. The wiki received a large volume of traffic year-round, with the most dramatic peak in visitation corresponding to timing of the fall semester. Popularity of the wiki grew with time as evidenced in the increase in many key website traffic variables from the Fall 2011 semester to the Fall 2012 semester. In a usage survey, most medical students reported awareness of the wiki as a resource (88%) and used it most often during the Human Body block (gross anatomy) of the preclinical years of the curriculum. A weak positive correlation was observed between willingness to edit the wiki and ability to edit the wiki.

The development and usage of the wiki underscores many principles of the constructivist theory of learning. Students chose to organize information in a format most meaningful to them by constructing pages that mirrored the curricular structure (year and blocks of body systems). Students also developed a rich folksonomy of useful websites and resources by providing website names, URLs, and descriptions. In one particular aspect, the CUSOM wiki served as a hybrid between traditional and constructivist models (Figure 1). An anatomy teacher in the medical curriculum added several websites and drawings (Figure 2B) to the Human Body block of the curriculum (Figure 2A). The ability of students to self-organize content enhanced the ability to find information efficiently. This was a major improvement over the previous Google Groups platform, in which older content was buried in the timeline and very difficult to retrieve.

Experiences documented in the literature from both successful and failed wikis have provided several key factors for establishing a successful and self-sustaining wiki. It is necessary to have an initial set of content and a user-friendly explanation of how to get started on adding content, a method of reassuring users that their content will be valued, and thorough testing and rapid response to technical difficulties: Cole [19] documented a failed attempt to incorporate a wiki into an undergraduate course on information systems, in which students frequently cited technical difficulty, lack of time, lack of interest, and hesitancy to be the first to post content as reasons for not editing the course wiki, which received zero contributions. Jalali et al [20] attempted to incorporate a student-created wiki into an undergraduate medical curriculum, but were not successful due to similar reasons as the aforementioned attempt-focus group. Comments included difficulty accessing the wiki, lack of content the users were looking for, and participant lack of confidence in their own knowledge to contribute. Although the CUSOM wiki has been reasonably successful, many of the same comments were echoed in this survey. Frequent reasons for not editing the wiki included unfamiliarity with the site, a difficult interface, or uncertainty about the value of their contributions. These barriers overlap with those reported in a study of emergency room residents’ beliefs about contributing to an online collaborative slideshow [18].

When asked to rate certain aspects of the wiki, some group differences emerged between the classes of medical students. Substantial differences were noted between upper-year (MS3 and MS4) and lower-year (MS1 and MS2) students in the perceived importance of the wiki in the preclinical and clinical years (Table 2). The lower ratings of the upper-year students (*P*<.001) may be due to their unfamiliarity with the wiki because it was not available to them at the onset of their curriculum. The lower-year students would have been aware of the wiki during their preclinical years and then further referenced it during their clinical education. Overall, the rating of perceived importance of the wiki fell on the lower end of the Likert scale for both preclinical (mean 2.73, SD 1.25 out of 5.00) and clinical (mean 1.88, SD 1.12) years. The greater response rates of the classes of 2013 and 2014 (46% and 36%) most likely lowered the overall averages. Other subtle, yet significant, differences were seen in the ease of finding and willingness to contribute content. The classes of 2015 and 2016 found it easier to find content than their more senior counterparts. This is perhaps due, again, to their familiarity with navigating the wiki beginning in their first or second year of study. The greater perceived importance of the wiki may be reflected in the greater willingness of the classes of 2015 and 2016 to contribute content to the resource. Two variables did not yield significant groups differences (ie, *P*>.05): (1) ease of making changes and (2) confidence of adding content. Although there was a trend toward a greater ease of making changes by the classes of 2013 and 2014, a very limited number of response to this survey item (n=20) likely unpowered our ability to detect any true differences. The rating of confidence across each class was very close to the middle of the Likert scale (3.00), which could be viewed positively and account for the successful development of the wiki with potential improvement in the presence of early formal guidance on adding content.

If the wiki is migrated to an on-campus server, then there will be an opportunity to significantly redesign the wiki structure to better conform to guidelines of good user interface. According to Sandars and Lafferty [21], visual design, consistency, accessibility, interactivity, and many other factors should be considered for e-learning resources in medical education. As part of the redesign process, focus groups and usability testing could be leveraged to create a standardized and easy-to-use layout to replace the existing layout, which evolved organically with the wiki and has been challenging to adapt to certain topics.

The survey results revealed several interesting trends. Overall, they show that similar to many learning tools, the CUSOM wiki is useful to some but not others, and depends greatly on individual learning style. For example, students who spend more time using digital study resources unsurprisingly consider the wiki more useful. Many students found the learning objective content to be helpful, but found it difficult to locate the content they needed or were frustrated by the number of embedded documents, which were harder to edit. Additional features, such as a template that makes it easier to arrange learning objectives or a small committee of appointed editors, could address some of these shortcomings, whereas others may have to wait for a complete redesign.

### Limitations

Major limitations of this study include a poor survey response rate (26%). In addition, responses were mostly from the classes of 2013 and 2014 (79% of respondents) and this likely introduced bias into the results from the survey. The usage survey in our study was constructed and reviewed by the authors for content validity. The survey quality could have been improved had reliability been tested (eg, Cronbach alpha) and validity demonstrated (eg, Kendall tau). Only responses from completed surveys were included in the analysis. Several survey items only had the end point of response options labeled, which left the meaning of unlabeled options open to respondent’s interpretation. This ambiguity may have introduced measurement error. Our use of a convenience sample was also a limitation of this study. Detailed demographic data for the survey respondents were not collected. Although we suspect our cohort of medical students is similar to those of other institutions, demographic data would assist in determination of generalizability. Objective data about how students actually used the wiki (eg, usability testing) were not gathered. In addition, implementation of a theory-based approach would also assist in understanding the barriers to using and contributing to a wiki in medical education.

### Future Work

Possible areas for future research on the wiki include repetition of the survey with future classes, investigation of the effect of requiring students to contribute, and—if the wiki is moved to a password-protected campus server—analysis of usage data at the level of individual users. Future attempts to engage students in editing the wiki will need to utilize multiple methods including a simple tutorial to reduce technical barriers, increased use of page templates to ensure user and editor-friendly pages, and possibly a curriculum component requiring students to edit the wiki to increase student buy-in and comfort. As indicated by the wide range of answers, there are users throughout the range of willing and able, unwilling and unable, willing but unable, and unwilling but able. Therefore, no single strategy will suffice to increase engagement. However, it is advisable to reduce the potential number of interventions to only those that are theory-based. Archambault et al [22] provide an excellent example of a theory-based approach in the field of medical education.

### Conclusions

This study details the creation of a medical curriculum-specific wiki, which was led by students. The wiki received a high volume of Web traffic that grew over time and was reported to be an important resource during preclinical and clinical years by students exposed during their first and second year of medical school.

## Appendix

Multimedia Appendix 1 The text of the survey distributed to all four current classes in the School of Medicine (n=640 students; Classes of 2013 to 2016).

## Abbreviations

CUSOM: University of Colorado School of Medicine
