# Supplementary material for: Taking Constructivism One Step Further: Post Hoc Analysis of a Student-Created Wiki
Source: JMIR Med Educ. 2018 Jun 14;4(1):e16. doi: 10.2196/mededu.9197 (PMC6024102; doi:10.2196/mededu.9197)
Supplement: Multimedia Appendix 1 [file mededu_v4i1e16_app1.pdf]

Appendix A

CUSOM Student Wiki Survey

1. What is your class year?
    - 2013
    - 2014
    - 2015
    - 2016

2. For approximately what percent of your studying do/did you use electronically accessed resources (computer, tablet, smartphone, etc) in your pre-clinical (first and second) years?
    - 0-25%
    - 26-50%
    - 51-75%
    - 76-100%

3. If applicable, for approximately what percent of your studying do/did you use electronically accessed resources (computer, tablet, smartphone, etc) in your clinical (third and fourth) years?
    - 0-25%
    - 26-50%
    - 51-75%
    - 76-100%

4. Prior to taking this survey, were you aware of the CUSOM Wiki (denversom.wikispaces.com)?
    - Yes
    - No

Questions for those who have used the site

5. How did you hear about the CUSOM Wiki?
    - From a classmate
    - From a professor or administrator
    - From someone in another class year
    - Internet search
    - From another website
    - Don't remember

6. Overall, how important is/was the Wiki for your education in your pre-clinical years?
    - 1 – Not important at all
    - 2
    - 3
    - 4
    - 5 – Essential

7. During which block in the pre-clinical years did you use the Wiki most?
- Human Body
- Molecules to Medicine
- Blood and Lymph
- Disease and Defense
- Cardiovascular, Pulmonary and Renal
- Nervous System
- Digestive, Endocrine and Metabolic Systems
- Infectious Disease
- None/Not applicable

8. During the block you used the Wiki the most, how often did you access the Wiki?
- Did not use for any block
- Monthly
- Weekly
- Several times per week
- Daily

9. If applicable, overall, how important is/was the Wiki for your education in your clinical years?
- 1 – Not important at all
- 2
- 3
- 4
- 5 – Essential

10. Please comment on any blocks or rotations where the Wiki was especially helpful, if any.
- [Open field response]

11. Please comment on any blocks or rotations where the Wiki could have been more helpful, and how.
- [Open field response]

12. How easily are you able to find the content you are looking for?
- 1 – Very difficult
- 2
- 3
- 4
- 5 – Very easy

13. Have you ever contributed content to the CUSOM Wiki, either directly or by asking someone else to add it?
- Yes, did it myself
- Yes, sent it to someone else
- No

14. If you have edited the Wiki before, how easy did you find it to make changes?

1 – Very difficult
2
3
4
5 – Very easy

15. If applicable, please comment on any specific challenges you have encountered using or editing the Wiki

[Open field response]

Questions for everyone

16. How willing will you be to contribute content to the Wiki in the future?

1 – Not at all
2
3
4
5 – Very willing

17. How confident are you that you could add content if you wanted to?

1 – Not at all
2
3
4
5 – Very confident

18. If you feel unable and/or unwilling to contribute to the Wiki, please describe your reasons

[Open field response]

19. Have you ever edited an article on any wiki other than the CUSOM Wiki (e.g., Wikipedia)

Yes
No

20. Additional Comments:

[Open field response]
